# Heterozygous *Vangl2^Looptail^* mice reveal novel roles for the planar cell polarity pathway in adult lung homeostasis and repair

**DOI:** 10.1242/dmm.028175

**Published:** 2017-04-01

**Authors:** Thanushiyan Poobalasingam, Laura L. Yates, Simone A. Walker, Miguel Pereira, Nina Y. Gross, Akmol Ali, Maria Kolatsi-Joannou, Marjo-Riitta Jarvelin, Juha Pekkanen, Eugenia Papakrivopoulou, David A. Long, Mark Griffiths, Darcy Wagner, Melanie Königshoff, Matthew Hind, Cosetta Minelli, Clare M. Lloyd, Charlotte H. Dean

**Affiliations:** 1 Inflammation Repair and Development Section, National Heart and Lung Institute, Imperial College London, London SW7 2AZ, UK; 2 Respiratory Epidemiology, Occupational Medicine and Public Health, National Heart and Lung Institute, Imperial College London, London SW3 6LR, UK; 3 Developmental Biology and Cancer Unit, UCL Institute of Child Health, London WC1N 1EH, UK; 4 Department of Epidemiology and Biostatistics, MRC–PHE Centre for Environment & Health, School of Public Health, Imperial College London, London SW7 2AZ, UK; 5 Center for Life Course Epidemiology, Faculty of Medicine, P.O. Box 5000, University of Oulu, Oulu FI-90014Finland; 6 Biocenter Oulu, P.O. Box 5000, Aapistie 5A, University of Oulu, Oulu FI-90014, Finland; 7 Unit of Primary Care, Oulu University Hospital, Kajaanintie 50, P.O. Box 20, Oulu FI-90220, Finland; 8 National Institute for Health and Welfare, Living Environment and Health Unit, Kuopio FI-70701, Finland; 9 University of Helsinki, Department of Public Health, Helsinki FI-00014, Finland; 10 National Institute for Health Research (NIHR) Respiratory Biomedical Research Unit at the Royal Brompton & Harefield NHS Foundation Trust and Imperial College, London SW3 6NP, UK; 11 Comprehensive Pneumology Center, Helmholtz Center Munich, Ludwig Maximilians University Munich, Munich 81377, Germany; 12 Department of Respiratory Medicine, Royal Brompton and Harefield NHS Foundation Trust, London SW3 6NP, UK; 13 Mammalian Genetics Unit, MRC Harwell Institute, DidcotOX11 0RD, UK

**Keywords:** Vangl2, Planar cell polarity, Lung disease, Lung homeostasis, Tissue repair, Cytoskeleton

## Abstract

Lung diseases impose a huge economic and health burden worldwide. A key aspect of several adult lung diseases, such as idiopathic pulmonary fibrosis (IPF) and chronic obstructive pulmonary disease (COPD), including emphysema, is aberrant tissue repair, which leads to an accumulation of damage and impaired respiratory function. Currently, there are few effective treatments available for these diseases and their incidence is rising. The planar cell polarity (PCP) pathway is critical for the embryonic development of many organs, including kidney and lung. We have previously shown that perturbation of the PCP pathway impairs tissue morphogenesis, which disrupts the number and shape of epithelial tubes formed within these organs during embryogenesis. However, very little is known about the role of the PCP pathway beyond birth, partly because of the perinatal lethality of many PCP mouse mutant lines. Here, we investigate heterozygous *Looptail* (*Lp*) mice, in which a single copy of the core PCP gene, *Vangl2*, is disrupted. We show that these mice are viable but display severe airspace enlargement and impaired adult lung function. Underlying these defects, we find that *Vangl2^Lp/+^* lungs exhibit altered distribution of actin microfilaments and abnormal regulation of the actin-modifying protein cofilin. In addition, we show that *Vangl2^Lp/+^* lungs exhibit many of the hallmarks of tissue damage, including an altered macrophage population, abnormal elastin deposition and elevated levels of the elastin-modifying enzyme, *Mmp12*, all of which are observed in emphysema. *In vitro*, disruption of *VANGL2* impairs directed cell migration and reduces the rate of repair following scratch wounding of human alveolar epithelial cells. Moreover, using population data from a birth cohort of young adults, all aged 31, we found evidence of an interactive effect between *VANGL2* and smoking on lung function. Finally, we show that PCP genes *VANGL2* and *SCRIB* are significantly downregulated in lung tissue from patients with emphysema. Our data reveal an important novel role for the PCP pathway in adult lung homeostasis and repair and shed new light on the genetic factors which may modify destructive lung diseases such as emphysema.

## Abstract (precis)

**Summary:** Manipulating the PCP pathway may provide new approaches to treat damaged lung tissue.

## INTRODUCTION

Globally, the burden of lung disease is enormous. For example, chronic obstructive pulmonary disease (COPD), which includes emphysema, is predicted to become the third leading cause of death worldwide by 2020 (López-Campos et al., 2016) and idiopathic pulmonary fibrosis (IPF) has a conservative incidence of 3-9 per 100,000 per year (Hutchinson et al., 2015). A key aspect of several adult lung diseases, including COPD, IPF and acute lung injury (ALI), is aberrant tissue repair, which culminates in declining lung function, tissue damage, and frequently, respiratory failure (Chilosi et al., 2013; Murray, 2012). Despite the substantial burden of degenerative lung diseases, there are currently no effective treatments available to modify repair and regeneration of damaged tissue. It has recently become apparent that adult mammalian lungs have an innate capacity for repair (Uhl et al., 2015; Butler et al., 2012). Therefore, identification of signalling pathways employed during development that are also capable of contributing to tissue repair, may lead to novel regenerative/repair approaches to treat lung disease (Kotton and Morrisey, 2014).

Lung development consists of several distinct phases; initially, the network of airways is generated via branching morphogenesis. Subsequently, the gas-exchanging region forms from the distal ends of small airways by thinning of interstitial tissue and widening of airspaces followed by formation of saccules that mature into alveoli (Herriges and Morrisey, 2014). The actin cytoskeleton has a critical role in morphogenesis by mediating organ shape and coordinated cell behaviours, including collective cell migration. Recently, the planar cell polarity (PCP) pathway has emerged as a critical regulator of tissue morphogenesis during embryonic development by directing actin cytoskeleton dynamics (Yates et al., 2010b; Munoz-Soriano et al., 2012; Wallingford, 2012). The PCP pathway is one of several signalling cascades that can be induced when a Wnt ligand binds to a transmembrane Frizzled receptor (Niehrs, 2012).

Previously, we have demonstrated in mouse embryos that homozygous mutations in several PCP pathway genes lead to impaired morphogenesis and disruption of lung and kidney development (Yates et al., 2010a,b). However, the role of the PCP pathway in the adult is almost entirely unknown. This is due, in part, to perinatal lethality caused by homozygous mutations of many PCP genes in mouse (Kibar et al., 2001; Murdoch et al., 2001). Here, we demonstrate that adult *Vangl2^Lp/+^* mice are viable but display many of the phenotypes observed in emphysema. Our data reveal that PCP pathway disruption perturbs both adult lung structure and function and adversely affects tissue repair in mouse and human lungs.

## RESULTS

### Embryonic and adult *Vangl2^Lp/+^* mice display disrupted lung architecture

**Fig. 1.**
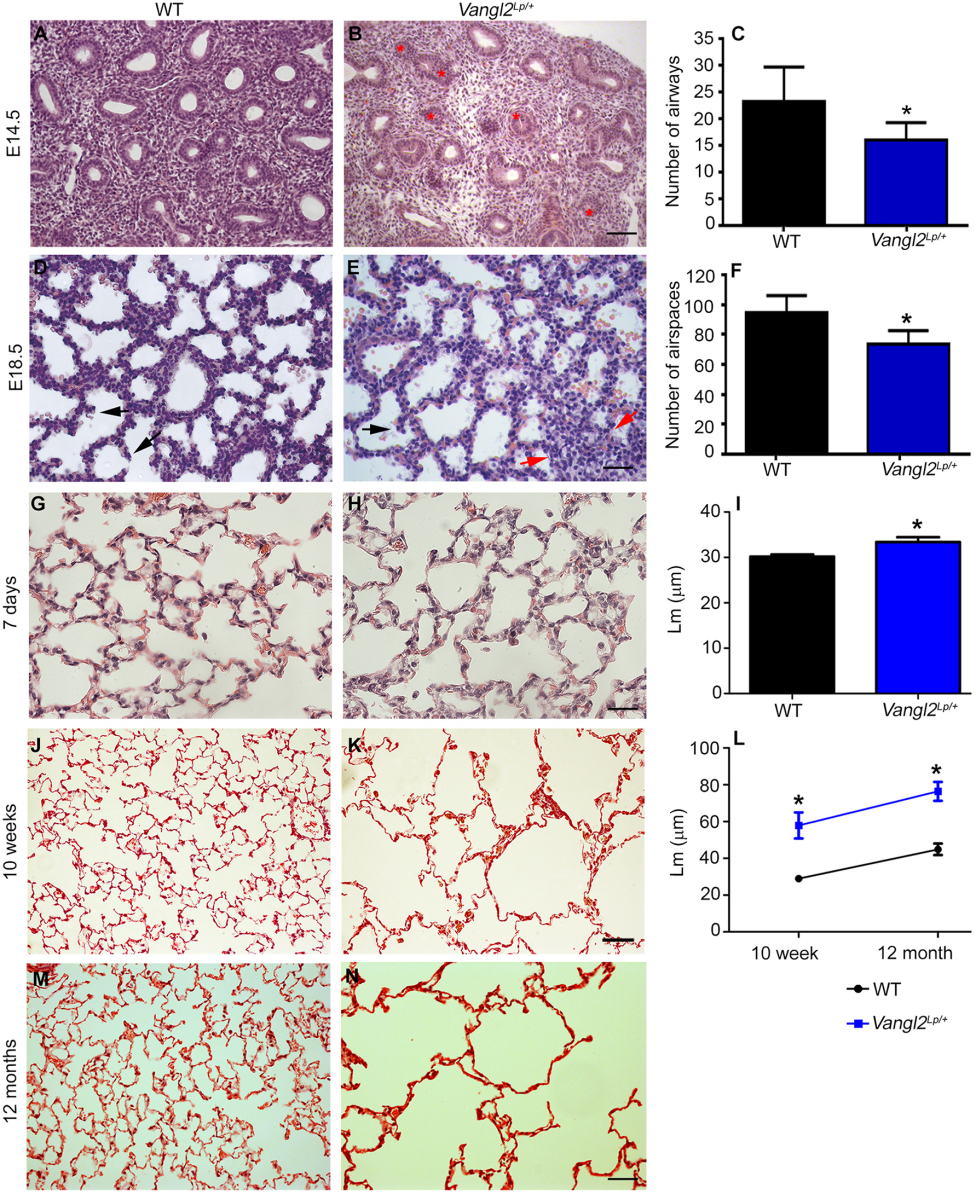
**A heterozygous Vangl2 *Looptail* mutation leads to altered structure in both embryonic and adult lungs.** H&E staining of lungs from embryonic (A,D) WT and (B,E) *Vangl2^Lp/+^* mutants at (A,B) E14.5 and (D,E) E18.5. (B) E14.5 *Vangl2^Lp/+^* showed many narrow or closed lumens (red asterisks). (C) *Vangl2^Lp/+^* E14.5 mice had a significant reduction in number of airways (16±0.87) compared with WT littermates (23.26±1.71; mean±s.e.m., *n*=3 per genotype; Student's *t*-test, *P<0.05). (E) E18.5 *Vangl2^Lp/+^* shows heterogeneous architecture with some areas appearing similar to WT mice with septation occurring (black arrow), whereas certain regions contain dense cells (red arrows). (F) E18.5 *Vangl2^Lp/+^* mice have a significant reduction in airway number (73.18±2.84) compared with WT littermates (94.36±3.48; *n*=3 per genotype; Student's *t*-test, *P<0.05). H&E staining of (G,H) 7-day-, (J,K) 10-week- and (M,N) 12-month-old mouse lungs shows enlarged alveolar spaces in (H,K,N) *Vangl2^Lp/+^* mutants compared with (G,J,M) WT littermates. (I) Quantification of the mean linear intercept (*L*_m_) revealed a significant increase in *L*_m_ in *Vangl2^Lp/+^* lungs (33.40±1.07 μm) compared with WT littermates at 7 days of age (30.18±0.48 μm; WT, *n*=4 and *Vangl2^Lp/+^*, *n*=3; Student's *t*-test, *P<0.05). (L) Similarly, *Vangl2^Lp/+^* lungs have increased *L*_m_ at 10 weeks (57.88±7.05 μm; *n*=5 per genotype; Student's *t*-test, *P<0.05) and 12 months (76.45±5.12 µm; *n*=3 per genotype; Student's *t*-test, *P<0.05) compared with WT littermates (28.98±0.46 μm and 44.90±3.14 µm, respectively). Scale bars: 50 µm (A,B), 12.5 µm (D,E), 23 µm (G,H), 47 µm (J,K) and 63 µm (M,N).

Homozygous *Vang2^Lp^* mice die at birth from neural tube defects but heterozygotes survive (Murdoch et al., 2001). To establish whether the single copy of the *Lp* mutation present in heterozygotes affected lung structure, we examined embryonic *Vangl2^Lp/+^* lungs. In comparison to WT lungs where the majority of airways had open, centrally located lumens, in *Vangl2^Lp/+^* mice, many airways contained only narrow or closed lumens (Fig. 1A,B) and there was a significant reduction in airway number (16.0 per field at 20× magnification) compared with wild-type (WT) littermates (23.26 per field at 20× magnification) (Fig. 1C). Just prior to birth at embryonic day (E)18.5, WT lung sections showed characteristic thinning of epithelial layers and a corresponding increase in airspace volume (Fig. 1D); however, in *Vangl2^Lp/+^* lungs, tissue architecture was heterogeneous; some areas of tissue appeared similar to WT whilst other regions contained highly condensed groups of cells with no airspaces (Fig. 1E, red arrows). Septation events (Fig. 1D,E, black arrows) and airway numbers were also reduced (*Vangl2^Lp/+^* 73.18 vs WT 94.36) (Fig. 1F). These embryonic defects were similar, but milder, than those in embryonic *Vangl2^Lp/Lp^* lungs (Yates et al., 2010b).

In postnatal lungs, mild but visible differences in lung structure were evident between WT (Fig. 1G) and *Vangl2^Lp/+^* (Fig. 1H) mutants at postnatal day (P)7, and quantification of mean linear intercept (Lm), showed a significant increase in *Vangl2^Lp/+^* mice (Fig. 1I). At 10 weeks of age, a time when bulk alveologenesis is complete, compared with WT (Fig. 1J), adult *Vangl2^Lp/+^* lungs displayed strikingly enlarged alveolar spaces, resembling emphysematous lung architecture (Fig. 1K); the mean linear intercept (*L*_m_) was significantly increased, at 57.88 µm in *Vangl2^Lp/+^*compared with 28.98 µm in WT (Fig. 1L). This abnormal lung structure persisted as the mice aged, and at 12 months, alveolar spaces remained significantly enlarged in *Vangl2^Lp/+^* (76.45 µm) compared with WT (44.90 µm) (Fig. 1L-N). As expected, *L*_m_ continued to increase with age in both WT and *Vangl2^Lp/+^*. However, the difference in *L*_m_ between WT and *Vangl2^Lp/+^* mice was similar at 10 weeks and 12 months, rather than diverging with age (Fig. 1L).

Development of the epithelial cell and vascular networks are tightly linked during alveologenesis, therefore disruption of one can affect the other. Despite considerable histological abnormalities in heterozygous lungs, comparison of WT and *Vangl2^Lp/+^* vasculature showed no significant differences in capillary network structure or expression levels of genes encoding vascular proteins at 10 weeks of age (Fig. S1A-C) or during embryonic development at E18.5 (Fig. S1D). To explore additional possible causes of the altered lung architecture in *Vangl2^Lp/+^* mice, we examined proliferation (Fig. S2A-C), apoptosis (Fig. S2D-F) and lung size (Fig. S2G-I) in adult mice. We also assessed differentiation in the lungs by immunostaining (Fig. S3); no significant changes were found in any of these parameters. Together, these data suggest that the structural alterations in adult *Vangl2^Lp/+^* mice arise from disrupted lung development rather than tissue destruction in the adult.

### Vangl2 is present in embryonic and adult mouse and human lungs and its localization is disrupted in embryonic *Vangl2^Lp/+^*

**Fig. 2.**
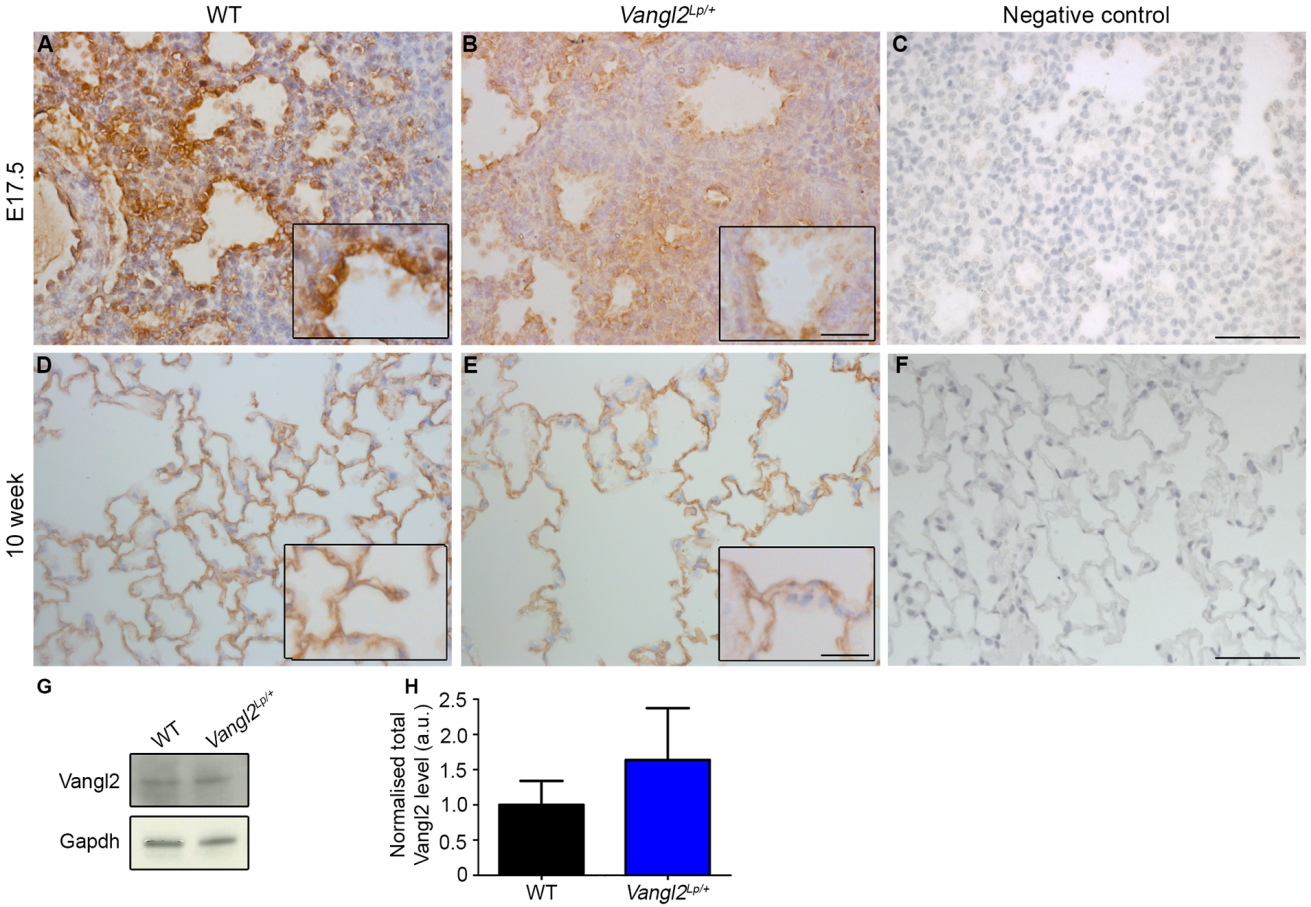
**The *Looptail* mutation results in mislocalization of Vangl2 in embryonic lungs.** Immunohistochemistry for Vangl2 (brown colour) and counterstaining with Haematoxylin (pale blue) in lungs of (A-C) embryonic and (D-F) adult mice. (A) In WT E17.5 lungs, Vangl2 is enriched in epithelial cells at apical surfaces of developing saccules. (B) In *Vangl2^Lp/+^* littermate lungs, apical enrichment of Vangl2 is reduced. (C) E17.5 lung negative control with primary antibody omitted. (D) In adult 10-week-old WT mice, Vangl2 staining is enriched at cell membranes. (E) A similar pattern is observed in adult *Vangl2^Lp/+^* alveolar epithelial cells. (F) 10-week-old adult lung negative control with primary antibody omitted. (G) Western blot for Vangl2 (70 kDa), and Gapdh (37 kDa) loading control, in lungs of 10-week-old WT and *Vangl2^Lp/+^* mice. (H) Quantification of protein showed no significant difference in Vangl2 levels between 10-week-old WT and *Vangl2^Lp/+^* lungs (mean±s.e.m., *n*=6 per genotype, two separate gels were run, each with lung lysate from three individual mice per genotype; Student's *t*-test, *P*=0.51). Scale bars: 45 µm (A-F) and 20 µm (insets).

We investigated how the *Lp* mutation affected Vangl2 protein in heterozygous lungs. At E17.5, immunostaining of WT lungs demonstrated that Vangl2 protein was present in both mesenchymal and epithelial cells with notable enrichment at apical membranes of epithelial cells (Fig. 2A). In *Vangl2^Lp/+^*, this apical enrichment was diminished and a more diffuse pattern of staining was observed adjacent to airspaces (Fig. 2B, control in C). In adult mouse lungs, Vangl2 staining was observed at the plasma membranes of alveolar epithelial cells (AECs) in both WT and *Vangl2^Lp/+^*, with no notable difference between genotypes (Fig. 2D,E, control in F). Western blotting demonstrated no significant difference in the level of Vangl2 between 10-week-old WT and *Vangl2^Lp/+^* whole lung lysates (Fig. 2G,H).

**Fig. 3.**
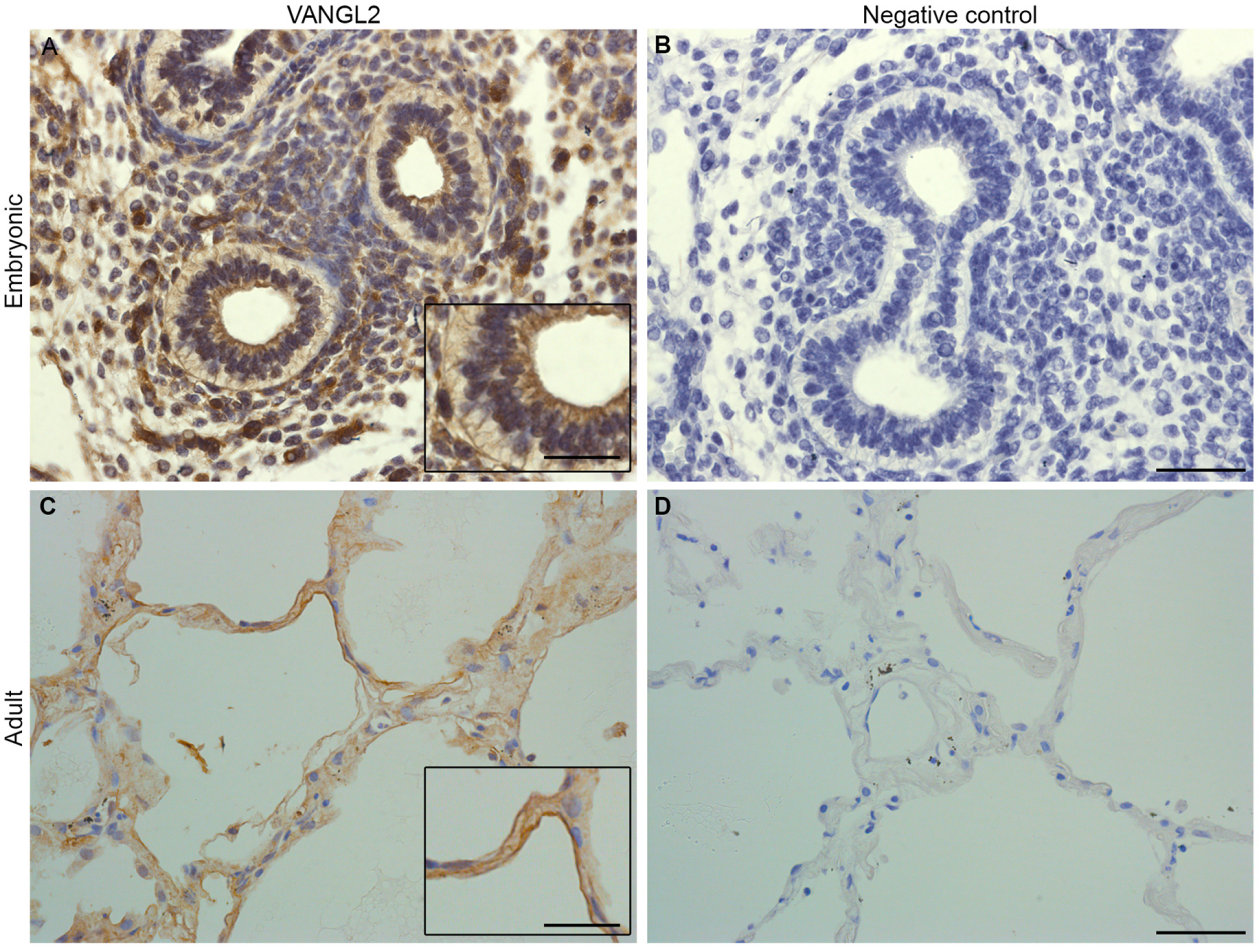
**VANGL2 protein is expressed in both embryonic and adult human lungs.** (A) Immunostaining for VANGL2 in embryonic human lungs at 10 weeks post conception shows prominent staining at the apical surfaces of developing airways (inset). (B) Human embryonic lung negative control with primary antibody omitted. (C) In human adult lungs, VANGL2 is localized to alveolar epithelial cells and is enriched at cell membranes. Inset highlights strong staining at the cell membrane. (D) Adult human lung negative control with primary antibody omitted. Scale bars: 32 µm (A-D) and 17 µm (insets).

Immunostaining of human lung sections showed similar VANGL2 localization with staining concentrated at the apical side of airways in pseudoglandular stage embryonic lung (Fig. 3A, control in B) and present at the cell membranes of AEC in normal adult human lung (Fig. 3C, control in D).

### Actin cytoskeleton distribution and regulation are altered in *Vangl2^Lp/+^* lungs

**Fig. 4.**
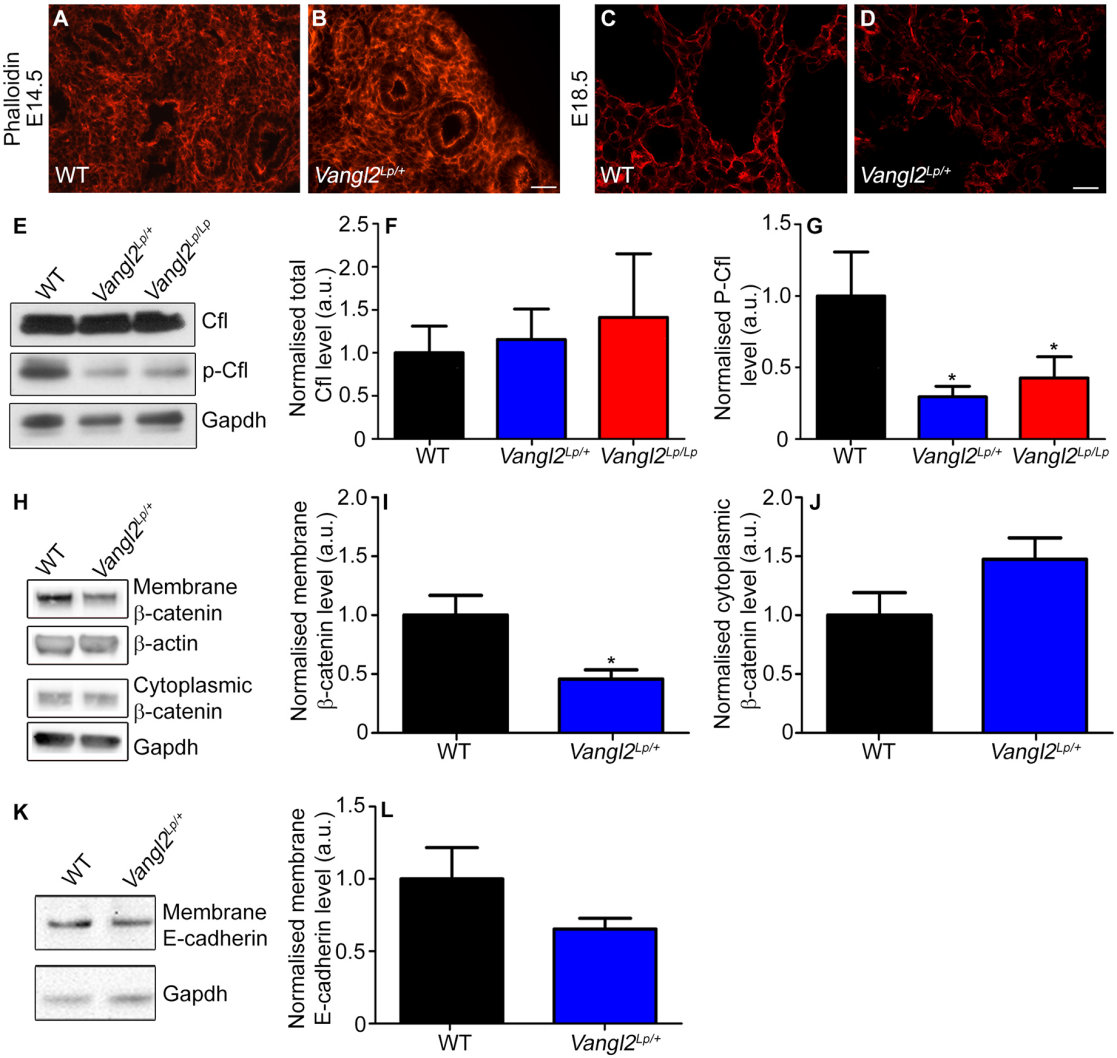
***Looptail* mutation disrupts actin cytoskeleton regulation.** (A-D) Phalloidin staining of filamentous actin distribution in (A,C) WT and (B,D) *Vangl2^Lp/+^* at (A,B) E14.5 and (C,D) E18.5. (E) Western blot of Cfl and p-Cfl (both 19 kDa) and Gapdh (37 kDa) in total protein lysate from E14.5 WT, *Vangl2^Lp/+^* and *Vangl2^Lp/Lp^* lungs. (F) Quantification of total Cfl in WT, *Vangl2^Lp/+^* and *Vangl2^Lp/Lp^* littermates, revealed no significant differences (mean±s.e.m., *n*=6 per genotype; 3 separate gels were run, with each gel containing 1 lane of lung lysate from each genotype. Each lane contained pooled lysate from two individual mice. Student's *t*-test, *P*>0.05). (G) p-Cfl levels are significantly reduced in both *Vangl2^Lp/+^* (70%) and *Vangl2^Lp/Lp^* (57%) lungs compared with WT littermates (*n*=6 per genotype; three separate gels were run, with each gel containing one lane of lung lysate from each genotype. Each lane contained pooled lysate from two individual mice; Student's *t*-test, *P<0.05). (H) Western blot of total β-catenin (92 kDa) in the cell membrane and cytosolic fractions of adult lungs, and β-actin (42 kDa) and Gapdh (37 kDa) loading controls. Quantification of β-catenin levels (I) in the membrane fraction showed 55% reduction in *Vangl2^Lp/+^* mice compared with WT controls (*n*=3 per genotype, with samples run on a single gel, Student's *t*-test, *P<0.05). (J) No significant difference was found in the cytosolic compartment (*n*=3 per genotype, with samples run on a single gel; Student's *t*-test, *P*>0.05). (K) Western blot of E-cadherin in the cell membrane fraction of adult lungs, and Gapdh (37 kDa) loading control. (L) Quantification of E-cadherin levels in the membrane fraction shows no significant difference between *Vangl2^Lp/+^* mice and WT controls (*n*=6 per genotype, two separate gels were run, each with lung lysate from three individual mice per genotype; Student's *t*-test, *P*>0.05). Scale bars: 25 µm (A,B) and 12.5 µm (C,D).

Because the PCP pathway can modulate actin cytoskeleton dynamics, we assessed filamentous actin (F-actin) distribution in *Vangl2^Lp/+^* lungs. At E14.5, there was no obvious difference in Phalloidin distribution between WT and *Vangl2^Lp/+^* (Fig. 4A,B). However, at E18.5, when the disordered architecture of heterozygous lungs was more distinct, there were marked differences in F-actin distribution between WT and heterozygotes. In WT, cortical actin was visible as a continuous ring around cells, closely associated with plasma membranes (Fig. 4C). However, in *Vangl2^Lp/+^* mice, cortical actin organization was frequently disrupted and discontinuous around cell perimeters so that the outline of individual cells could not be distinguished (Fig. 4D).

To explore the mechanisms underlying these changes in F-actin distribution, we investigated the key actin-remodelling factor, cofilin. We compared cofilin regulation in developing lungs from *Vangl2^Lp/+^* and *Vangl2^Lp/Lp^* mice at E14.5, a time point when it is possible to obtain both heterozygous and homozygous mutants and when extensive cell migration occurs. Total cofilin levels were not significantly altered in either of the *Vangl2* genotypes compared with WT littermates (Fig. 4E,F). However, there was a striking reduction in phosphorylated cofilin levels in *Vangl2^Lp/+^* (70%) and *Vangl2^Lp/Lp^* (57%) lungs compared with WT (Fig. 4E,G), indicating that a higher level of active (non-phosphorylated) cofilin was present in embryonic *Vangl2* mutant mice.

The actin cytoskeleton is closely associated with cell-cell adhesion. Therefore, we performed subcellular fractionation to isolate the membrane and cytoplasmic fractions and assessed the quantity of adherens junction proteins within each fraction. In the membrane fractions, β-catenin levels were significantly reduced by 55% compared with WT littermates (Fig. 4H,I). In the cytoplasmic fraction, a slight increase in β-catenin was detected in *Vangl2^Lp/+^* lungs (Fig. 4H,J) but this was not statistically significant and consistent with this finding, there was no difference in canonical Wnt/β-catenin signalling activity between WT and *Vangl2^Lp/+^* lungs (Fig. S4). In contrast to β-catenin, E-cadherin levels were not significantly altered between WT and *Vangl2^Lp/+^* membrane fractions (Fig. 4K,L).

### VANGL2 knockdown impairs cell migration

**Fig. 5.**
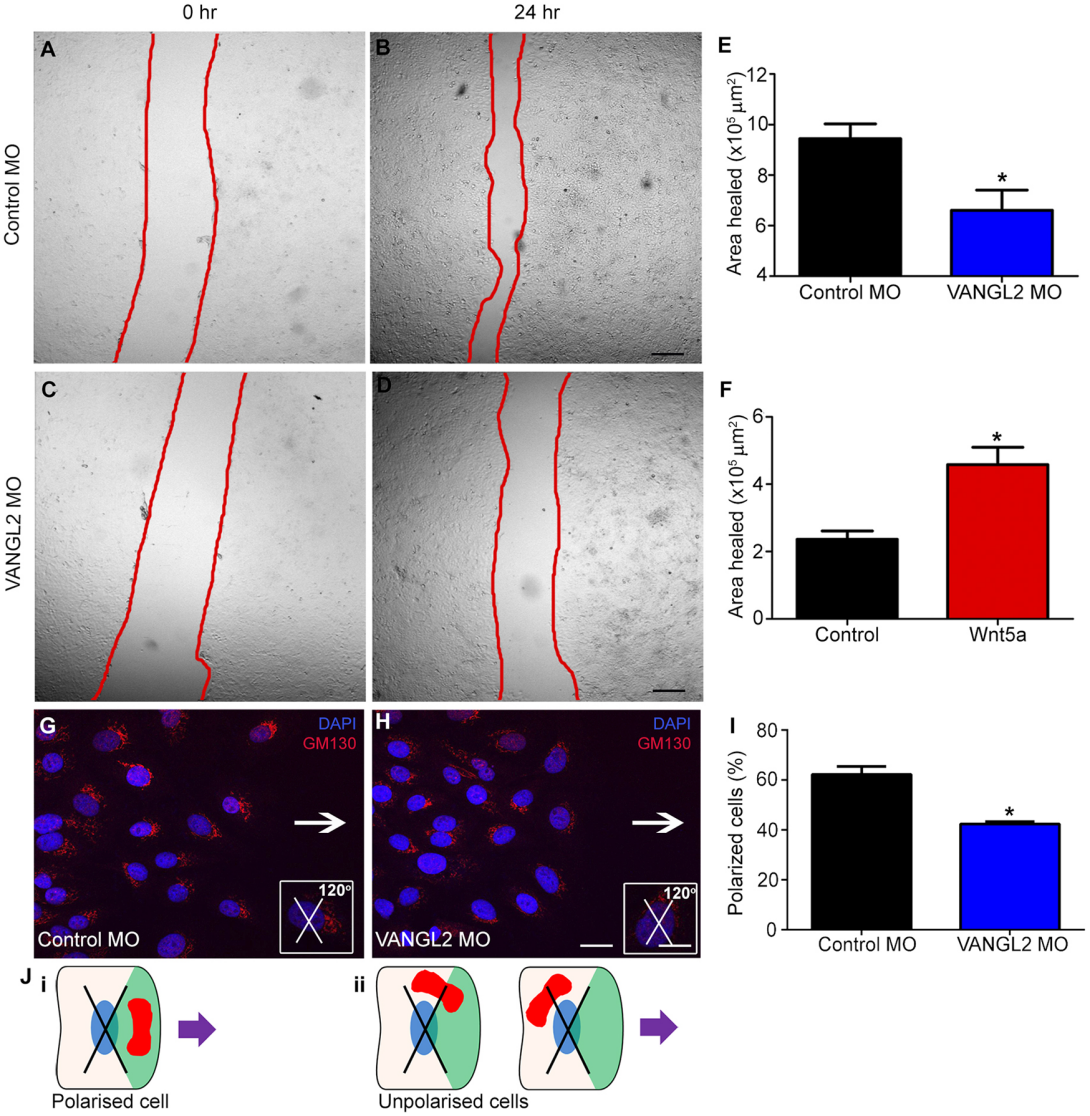
**VANGL2 knockdown in A549 cells leads to impaired wound healing.** (A-D) Representative images showing wound edges (red lines), in A549 cells treated with (A,B) control or (C,D) anti-VANGL2 morpholino oligonucleotide (MO), (A,C) 0 h and (B,D) 24 h post scratch. (E) VANGL2 knockdown led to a 30% reduction in the area healed at 24 h compared with controls (mean±s.e.m., *n*=5 per treatment, with at least three technical repeats for each condition per experiment; Student's *t*-test, *P<0.05). (F) A549 cells stimulated for 18 h with WNT5A showed a 94% increase in the rate of wound healing compared with controls following scratch injury (*n*=3 per treatment, with at least three technical repeats for each condition per experiment; Student's *t*-test, *P<0.05). (G,H) Immunofluorescence staining for the Golgi marker (GM130) to assess cell polarity during directed cell migration at the leading edge. Insets show crosses with a 120° arc facing the direction of travel (white arrow) placed on individual cell nuclei; cells with Golgi situated within that arc were classified as polarized. (I) Quantification of percentage of polarized cells showed a significant reduction following VANGL2 knockdown compared with controls (*n*=3 per treatment; Student's *t*-test, *P<0.05). (J) Schematic illustrating the position of the 120° arc on the nucleus (blue) of cells at the leading edge. Purple arrow shows the direction of migration. (i) Golgi (red) that are located within the forward-facing 120° arc (green quadrant) were classed as polarized, (ii) cells where either the whole or majority of the Golgi body lies outside the area 120° arc were classed as not polarized. Scale bars: 322 µm (A-D), 21 µm (G,H) and 11.3 µm (insets).

The changes to actin cytoskeleton present in *Vangl2^Lp/+^* prompted us to investigate whether VANGL2 modulation might affect lung cell migration and wound repair. We performed scratch assays in human alveolar epithelial cells (A549) and compared the rate of ‘wound-healing’ in control and VANGL2-knockdown cultures, using morpholino oligonucleotides (MO) to deplete VANGL2 protein. VANGL2 knockdown led to a 30% reduction in the area of wound healed at 24 h compared with controls (Fig. 5A-E). Conversely, stimulation of the PCP pathway by recombinant WNT5A, a ligand for the Wnt/PCP pathway (Komiya and Habas, 2008; Witze et al., 2008), showed accelerated cell migration leading to a 94% increase in the area of wound healed compared with controls by 18 h (Fig. 5F). MO knockdown efficiency was assessed by immunostaining (Fig. S5A,B and control in C) and a 48.8% reduction in staining levels was calculated following VANGL2 knockdown (Fig. S5D). A similar decrease in wound-healing efficiency was also observed following preliminary experiments with siRNA-mediated knockdown of VANGL2 (Fig. S6).

Collective cell migration is also important for efficient tissue repair, and as part of this process, cells adopt a uniform polarity to migrate in the same direction. As the PCP pathway has been shown to be important for directed cell migration in other cells/tissues, we assessed directed cell migration following VANGL2 knockdown, by quantifying the number of ‘polarized’ cells at the leading edge of the scratch wound using the Golgi marker GM130. Significantly fewer polarized cells, with Golgi on the side of the nucleus nearest to the wound, were present in VANGL2-knockdown cultures (Fig. 5G,H,J): 42.4% compared with 62.29% of cells in control MO cultures (Fig. 5I). These data show that depletion of VANGL2 impairs both the rate of wound repair and directed cell migration in human alveolar epithelial cells following injury.

### Lung function is reduced in *Vangl2^Lp^* heterozygotes

**Fig. 6.**
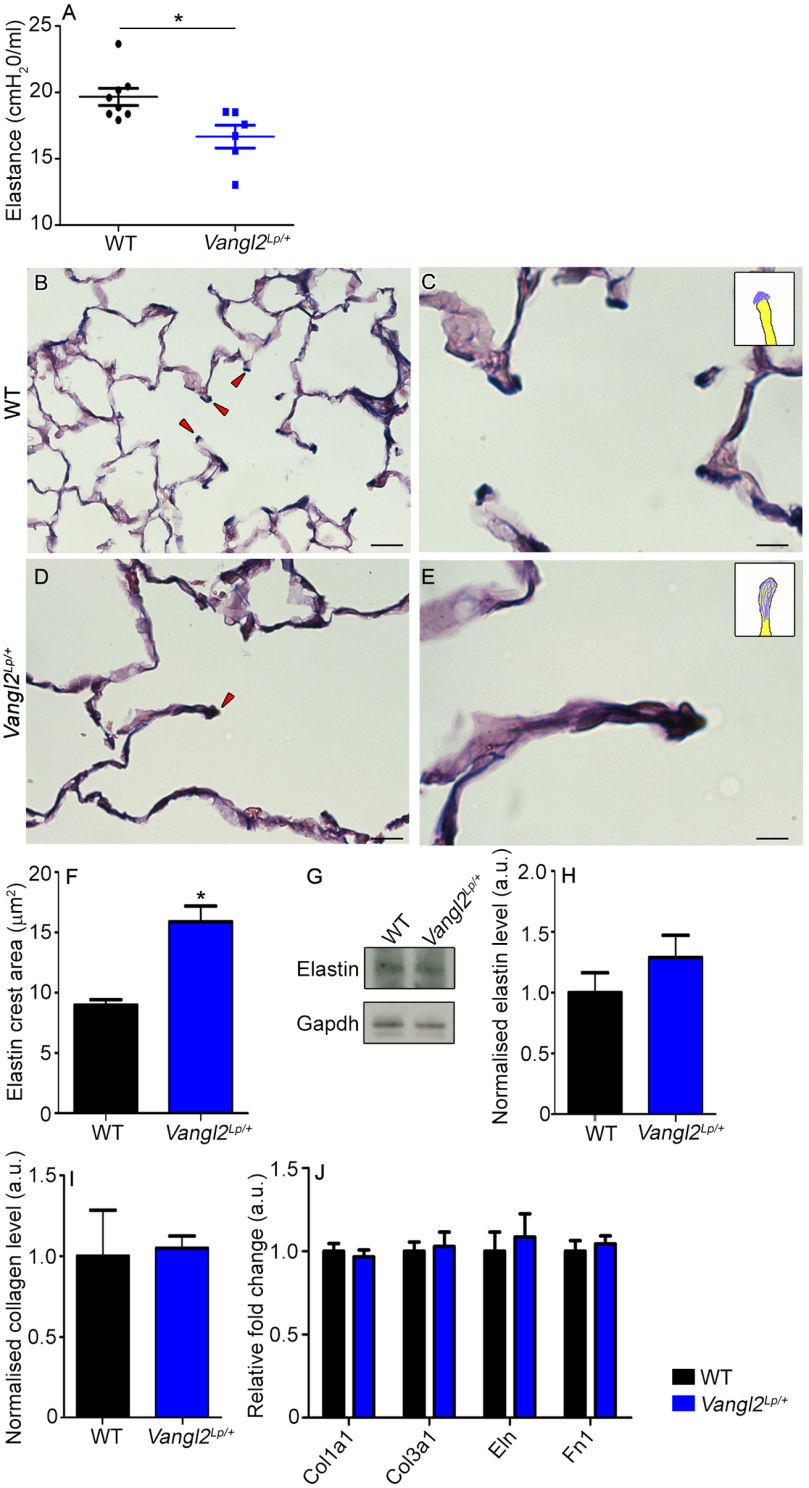
**Adult *Vangl2^Lp/+^* mice have altered lung function and disrupted elastin organization.** (A) Scatter plot showing lung function measurements in 10-week-old mice. *Vangl2^Lp/+^* mice show reduced elastance (16.66±0.856 cmH_2_O/ml) compared with WT littermates (19.67±0.651 cmH_2_0/ml) (mean±s.e.m., each point shows an individual mouse with data combined from two independent experiments; Student's *t*-test, *P<0.05). Miller's elastin staining of (B,C) WT and (D,E) *Vangl2^Lp/+^* 10-week-old lung sections highlights elastin (dark blue colour). Elastin deposition is prominent at the tips of alveolar crests (red arrowheads). Insets in C and E show a diagrammatic representation of the patterns of elastin deposition at alveolar crests. (F) The area of elastin at the tips of alveolar crests is increased in *Vangl2^Lp/+^* lungs (15.89±1.30 μm^2^) compared with WT littermates (8.89±0.45 μm^2^) (WT, *n*=4 and *Vangl2^Lp/+^*, *n*=5; Student's *t*-test *P<0.05). (G) Western blot of total elastin (60 kDa) in 10-week-old WT and *Vangl2^Lp/+^* littermates, and Gapdh (37 kDa) loading control. (H) Quantification revealed no significant difference in elastin protein (*n*=4 per genotype, with samples run on a single gel; Student's *t*-test, *P*=0.28). (I) Sircol assay for quantification of total collagen levels, in lungs of *Vangl2^Lp/+^* and WT littermates shows no significant difference (*n*=3 per genotype; Student's *t*-test, *P*=0.78). (J) No significant differences are observed in relative fold change of remodelling genes, *Eln*, *Col1a1*, *Col3a1* and *Fn1*, between adult *Vangl2^Lp/+^* and WT controls (WT, *n*=8 and *Vangl2^Lp/+^*, *n*=6; Student's *t*-test, *P*>0.05). Scale bars: 12.5 µm (B,D), 5 µm (C,E).

To determine whether the structural and cytoskeleton defects disrupt lung function in adult *Vangl2^Lp/+^* mice, we used the Flexivent system to compare 10-week-old WT and *Vangl2^Lp/+^* littermates. *Vangl2^Lp/+^* mice showed a significant decrease in elastance compared with WT littermates (16.66 vs 19.67 cmH_2_O/ml) (Fig. 6A). We then examined collagen and elastin in the lungs, both of which are central to the maintenance and integrity of normal lung structure and function. We compared elastin deposition at the tips of alveolar secondary crests, where it is thought to have a key role in septation. In WT lungs, elastin fibres were closely clustered together forming tight bundles in a small area at the very tip of secondary crests (Fig. 6B,C, illustrated in insert in C), whereas fibers were more widely dispersed in *Vangl2^Lp/+^* (Fig. 6D,E, illustrated in insert in E). Quantification of the area of elastin deposition at crest tips revealed a significant increase in *Vangl2^Lp/+^* mutants (15.89 µm^2^) compared with WT mice (8.98 µm^2^) (Fig. 6F). Total elastin levels were similar between WT and *Vangl2^Lp/+^* mice (Fig. 6G,H). Collagen levels were not altered in *Vangl2^Lp/+^* (Fig. 6I). No significant differences were detected in transcript levels of collagen, elastin and fibronectin between *Vangl2^Lp/+^* and WT (Fig. 6J). Moreover, we had previously determined that levels of Pdgfr-α, a factor associated with myofibroblasts during septation, were not significantly altered in *Vangl2^Lp/+^* (Fig. S1C,D), highlighting that the *Vangl2^Lp^* mutation appears to specifically affect elastin organisation.

### *Vangl2^Lp/+^* lungs exhibit phenotypes associated with tissue damage

**Fig. 7.**
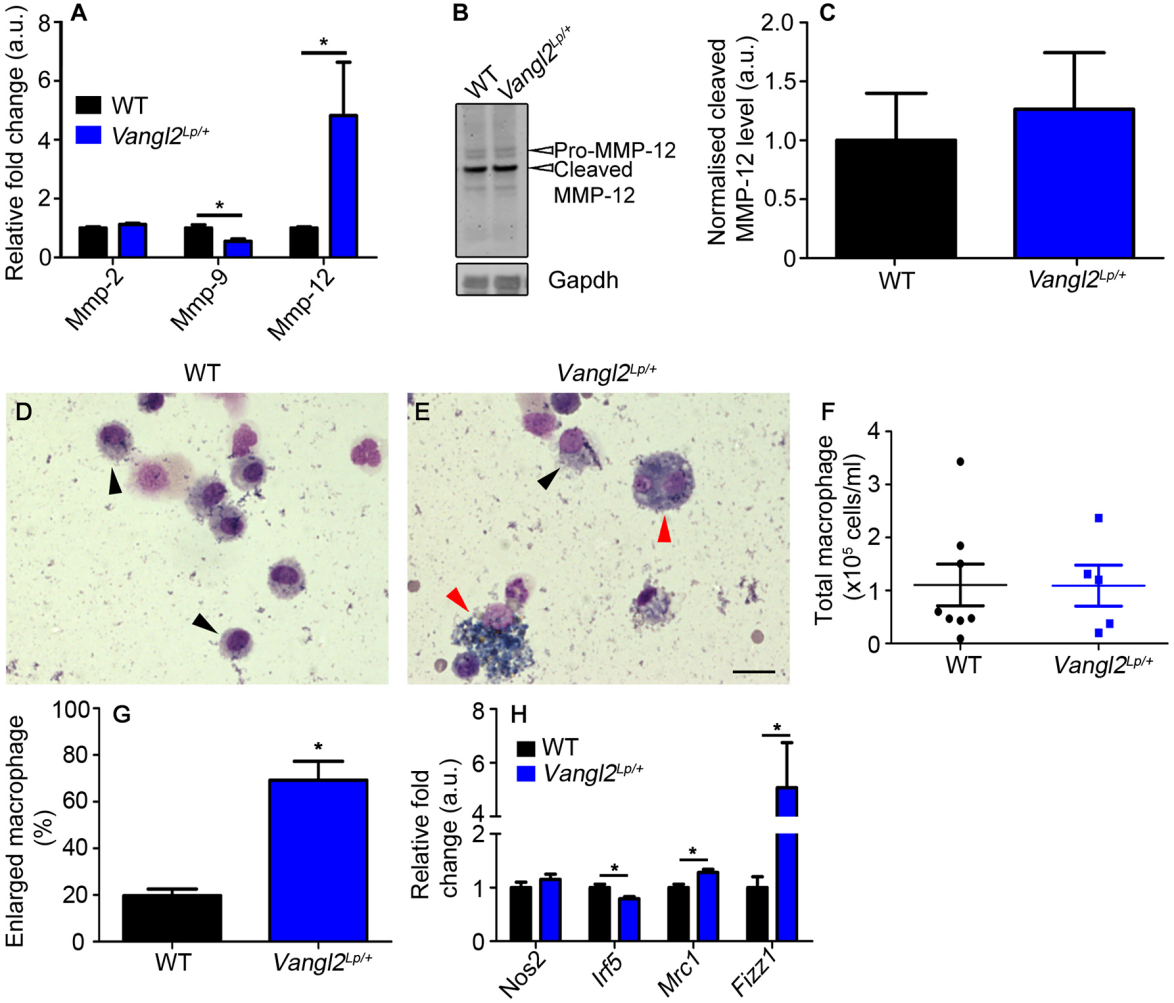
**Macrophage phenotype is altered in adult *Vangl2^Lp/+^* mice.** (A) qRT-PCR analysis for *Mmp2*, *Mmp9*, and *Mmp12*, revealed reduced *Mmp9* and increased *Mmp12* expression in adult *Vangl2^Lp/+^* mice compared with WT (mean±s.e.m.; WT, *n*=8 and *Vangl2^Lp/+^*, *n*=6; *P<0.05). (B) Western blot of Mmp12 showing ProMmp12 (54 kDa) and cleaved Mmp12 (45 kDa) in 10-week-old WT and *Vangl2^Lp/+^* littermates and Gapdh (37 kDa) loading control. (C) Quantification of the 45 kDa cleaved (active) Mmp12 protein levels revealed no significant difference (mean±s.e.m., *n*=3 per genotype. All samples were run on the same gel and a second gel was run with the same samples as a technical replicate; Student's *t*-test, *P*=0.69). Cytospin images from BALF of 10-week-old (D) WT and (E) *Vangl2^Lp/+^* mice showing normal (black arrowheads) and enlarged macrophages (red arrowheads). (F) Scatter plot showing total macrophage numbers in BALF of WT and *Vangl2^Lp/+^* littermates (WT, *n*=8 and *Vangl2^Lp/+^*, *n*=5 mice; Student's *t*-test, *P*>0.05). (G) *Vangl2^Lp/+^* mice have an increased percentage of enlarged macrophages in BALF compared with WT littermates (WT, *n*=8 and *Vangl2^Lp/+^*, *n*=7; Student's *t*-test, *P<0.05). (H) qRT-PCR analysis for *Nos2*, *Irf5*, *Mrc1* and *Fizz1* in 10- to 14-week-old mice showed increased M2 and reduced M1 marker transcript expression in *Vangl2^Lp/+^* lungs compared with WT littermates (WT, *n*=8 and *Vangl2^Lp/+^*, *n*=6; Student's *t*-test, *P<0.05). Scale bar: 11 µm (D,E).

Matrix metalloproteinase (MMP) enzymes are involved in remodelling of extracellular matrix (ECM) components such as elastin and collagen; therefore, we compared transcript levels of three key MMP enzymes, *Mmp2*, *Mmp9* and *Mmp12*, in WT and *Vangl2^Lp/+^* lungs by qRT-PCR. Levels of *Mmp12*, the macrophage-derived protease responsible for degrading elastin, were almost 5-fold higher in *Vangl2^Lp/+^* lung tissue compared with WT levels (Fig. 7A). *Mmp9* levels were significantly reduced whereas *Mmp2* was not altered (Fig. 7A). We quantified one of the major cleaved MMP12 protein forms in lung lysate but we did not detect a significant difference in this particular active form of MMP12 between *Vangl2^Lp/+^* and WT (Fig. 7B,C).

Mmp12 is predominantly macrophage-derived, therefore we examined macrophages in *Vangl2^Lp/+^* lungs. Analysis of bronchoalveolar lavage fluid (BALF) from WT and *Vangl2^Lp/+^* mice (Fig. 7D,E) showed no difference in total macrophage numbers (Fig. 7F); however, we noted a distinct population of enlarged, highly vacuolar macrophages in mutant BALF (Fig. 7E; red arrowheads). *Vangl2^Lp/+^* lungs contained a markedly increased proportion of these macrophages, 69.20% compared with 19.17% in WT littermates (Fig. 7G). Further analysis showed a significant increase in expression of the M2 tissue-remodelling-associated genes, *Mrc1* (mannose receptor 1) and *Fizz1* (found in inflammatory zone 1), concomitant with a decrease in expression of the M1 type marker *Irf5* (interferon regulatory factor 5) in *Vangl2^Lp/+^* lungs compared with WT littermates (Fig. 7H).

### Interaction of *VANGL2* polymorphisms with a damaging environmental insult (smoking) modifies human lung function

To investigate whether our findings might be relevant to human lung function, we assessed the effect of single nucleotide polymorphisms (SNPs) in the *VANGL2* gene on lung function in a population-based sample of 5139 young adults, all aged 31, from the Finnish NFBC1966 birth cohort study (Rantakallio, 1988). Since smoking represents an example of strong injurious insult to the lung in humans, we also investigated whether *VANGL2* SNPs could modify the negative effects of smoking on lung function.

We considered two measures of lung function, forced vital capacity (FVC), a spirometry parameter indicating lung restriction as well as lung size, and the ratio of forced expiratory volume in 1 s over FVC (FEV_1_/FVC ratio), an indicator of airway obstruction. We investigated the presence of interactions between smoking, defined in terms of pack-years smoked, and 14 SNPs in *VANGL2* on FVC and FEV_1_/FVC ratio. Application of elastic-net variable selection to linear regression models of FVC and FEV_1_/FVC ratio (see Materials and Methods) identified a significant SNP×smoking interaction for FVC, but not for FEV_1_/FVC. The SNP identified as interacting with smoking on FVC, rs4656907 (Table 1), lies in intron 3 of the *VANGL2* gene. The rs4656907 SNP is in linkage disequilibrium with a synonymous SNP located in exon 8 of *VANGL2*, rs17380127 (*r*^2^=0.74) − the same exon where the Looptail mutation is located in the mouse. In addition, publicly available eQTL data, searched using the GTEx portal (www.gtexportal.org), show an association of this SNP with decreased expression levels of *VANGL2* in thyroid tissue. The interaction effect of rs4656907 with smoking on FVC was −5.1 ml per allele and pack-year (95% confidence interval: −8.7 to −1.5; *P*=0.005). Interestingly, neither the SNP alone nor smoking alone had a significant effect on FVC (Table 1).

**Table 1.**
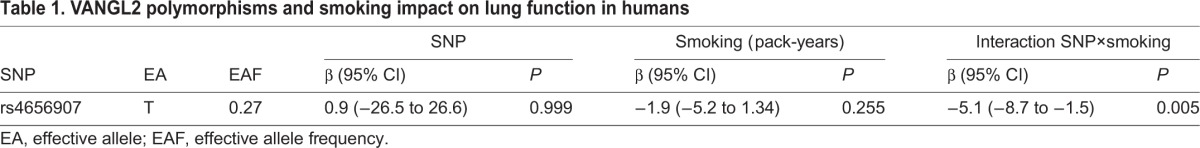
**VANGL2 polymorphisms and smoking impact on lung function in humans**

**Fig. 8.**
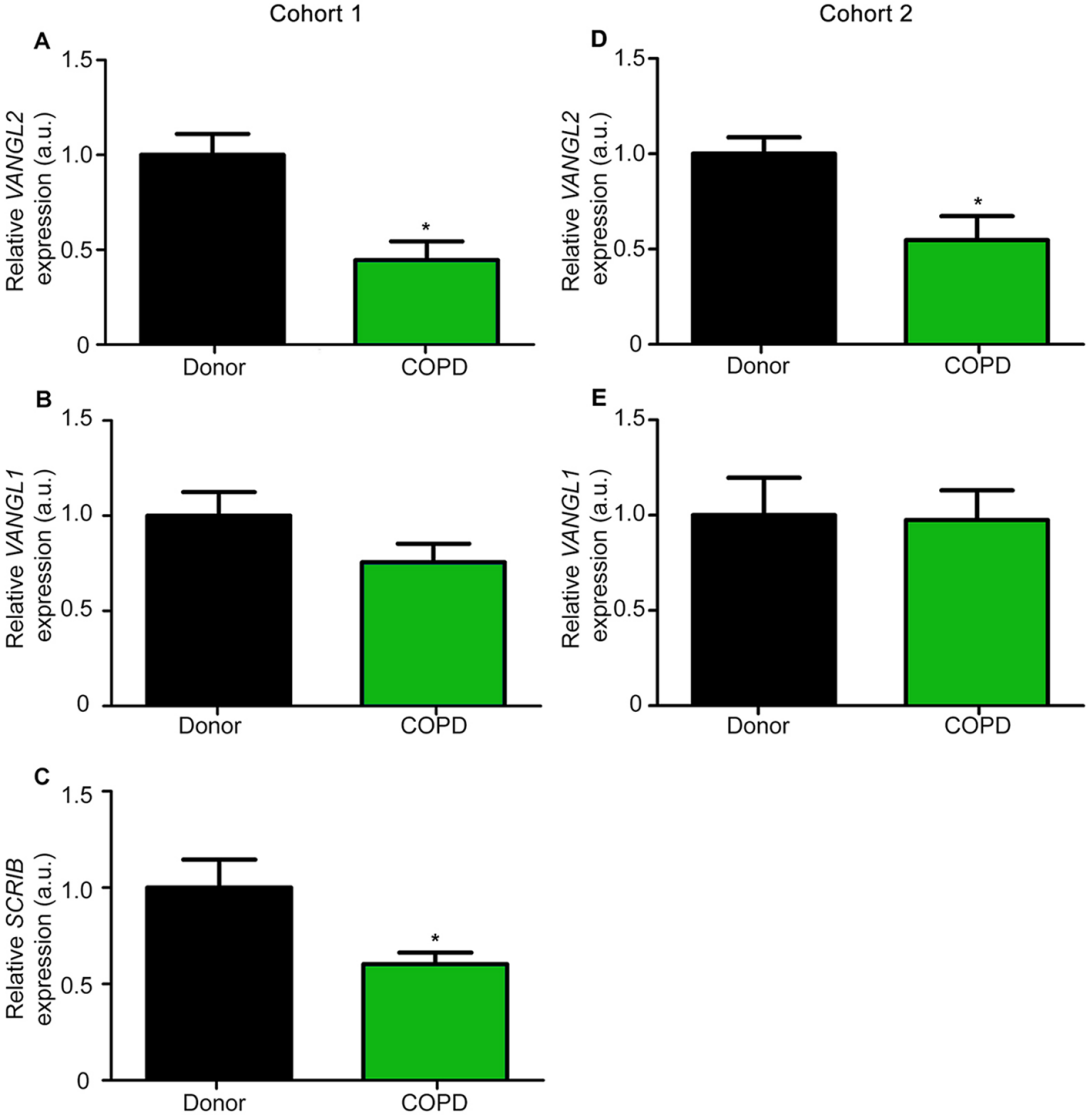
***VANGL2* and *SCRIB* expression are significantly downregulated in COPD patient lungs.** Relative gene expression of (A,D) *VANGL2*, (B,E) *VANGL1* and (C) *SCRIB*, in COPD patient lungs, compared with control lungs from (A-C) cohort 1 and (D,E) cohort 2; gene expression was normalized to *B2M* expression (mean±s.e.m., *n*=6 per group in cohort 1 and *n*=8 per group in cohort 2; Student's *t*-test, *P<0.05).

We also compared the levels of several PCP gene transcripts in lung tissue samples from healthy transplant donor controls and patients with COPD (where lung tissue damage is present) by qRT-PCR (cohort 1). *VANGL2* mRNA levels were considerably lower in COPD patients at less than half that detected in healthy controls (Fig. 8A). Transcript levels of the PCP gene *SCRIB*, were also significantly reduced in COPD patients (40% reduction; Fig. 8C). In contrast, levels of the *VANGL2* homologue, *VANGL1*, were unaltered in the COPD samples (Fig. 8B). Levels of *VANGL2* (Fig. 8D) and *VANGL1* (Fig. 8E) were quantified in a second patient cohort (cohort 2) and the findings from cohort 1 were confirmed.

## DISCUSSION

The PCP pathway has been extensively studied in embryonic development, but the consequences of these defects in adults are poorly understood, partly because of the perinatal lethality of murine homozygous PCP gene mutants (Copley et al., 2013; Juriloff and Harris, 2012). Apart from recent findings that podocyte-specific deletion of *Vangl2* leads to increased susceptibility to injury in adult mouse kidney following challenge (Rocque et al., 2014), and links between PCP gene dysfunction and cancer (Elsum et al., 2014; Hatakeyama et al., 2014), the role of the PCP pathway in adulthood is almost entirely unstudied. Given the known role of this pathway in actin cytoskeleton remodelling and collective cell migration during embryonic development, we hypothesized that the PCP pathway might be important for adult lung homeostasis and in tissue repair following injury. This study reveals that *VANGL2* polymorphisms can significantly alter lung structure and function, as well as modifying the capacity for tissue repair following injury.

### PCP signalling defects influence actin cytoskeleton polymerization and localization of β-catenin at the plasma membrane

**Fig. 9.**
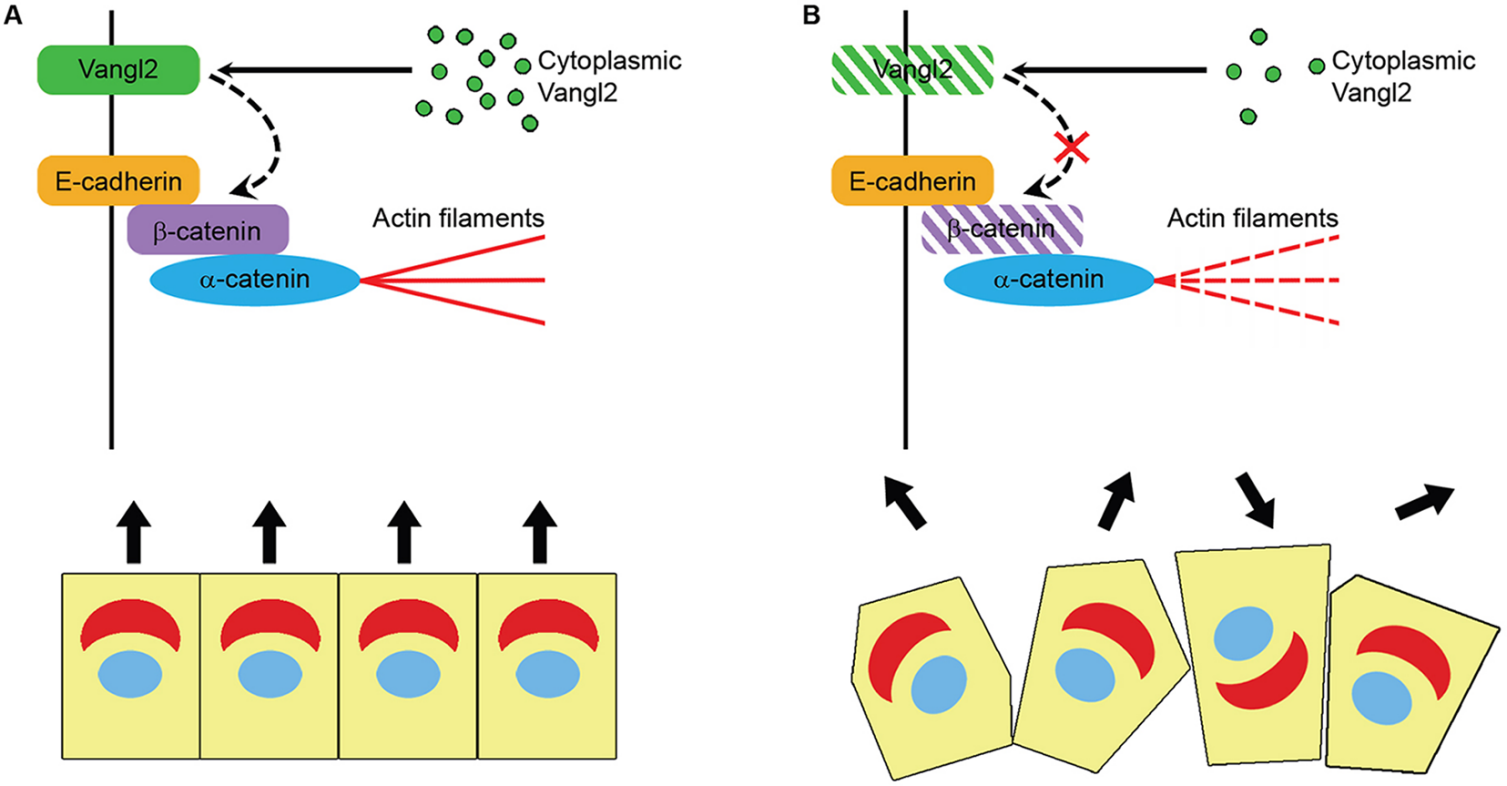
**Schematic summarizing the effects of Vangl2 modulation on lung epithelial cells.** (A) Under normal conditions, Vangl2 is packaged in vesicles within the Golgi prior to being transported to the plasma membranes. (B) Modulation of Vangl2 results in reduced levels of membrane-associated Vangl2 and β-catenin as well as disrupted actin cytoskeleton integrity and impaired cell migration following injury.

The *Looptail* mouse is a powerful tool for investigating the functions of Wnt/PCP signalling because the underlying mutation not only affects *Vangl2*, perturbing its trafficking to the cell membrane (Wansleeben et al., 2010; Yin et al., 2012; Merte et al., 2010), but it also affects additional PCP components such as Prickle2 (Pryor et al., 2014), Frizzled3 (Montcouquiol et al., 2006) and its homologue Vangl1 (Yin et al., 2012). Our study identifies actin cytoskeleton abnormalities in *Vangl2^Lp/+^* lungs, as well as dysregulation of cofilin. Cofilin is an actin-remodelling protein that has been associated with PCP signalling in embryogenesis (Bravo-Cordero et al., 2013; Mahaffey et al., 2013). In its active form, cofilin binds to and severs actin filaments, increasing the rate of depolymerization and recycling of actin filaments, a process that is necessary to drive cell migration (McGough et al., 1997). In addition to these changes in actin cytoskeleton activity, we also found a striking reduction in β-catenin specifically at the cell membranes of adult *Lp* heterozygote lungs. This is particularly interesting given that membrane-bound β-catenin links E-cadherin with the actin cytoskeleton and through this role, β-catenin is important to maintain proper actin filament structure and tension as well as cell-cell adhesion. Both actin cytoskeleton remodelling and rearrangement of cell adhesions are required for tissue repair, where cells must migrate to remodel the damaged area (Abreu-Blanco et al., 2012). Together with our findings that both VANGL2 and WNT5A modify the rate of epithelial wound-healing following scratch injury, these data illustrate that the PCP pathway can modify wound repair in adult lung (Fig. 9).

### The PCP pathway as a novel target for diseases involving aberrant tissue repair

Manipulation of Wnt signalling pathways is a promising avenue for novel therapies to target diseases involving aberrant tissue repair (Whyte et al., 2012). Uhl and colleagues recently demonstrated the feasibility that Wnt signalling manipulation can alter adult lung tissue architecture (Uhl et al., 2015), but to date, modulation of the PCP pathway has not been explored as a possible target to modify tissue repair. Our data indicate that the PCP pathway is important for tissue repair following human lung injury. In this study, we show that *VANGL2* and *SCRIB* are significantly downregulated in tissue from COPD patients compared with healthy controls. These findings are in agreement with a global analysis of altered gene expression levels between normal and COPD patient samples, which list a number of PCP genes that are significantly altered (Ezzie et al., 2012).

Our experimental findings are further strengthened by the results of a population-based study presented in this manuscript, which identifies a polymorphism in *VANGL2*, rs4656907, which in combination with an injurious insult (smoking), is associated with reduced lung function (FVC). For this study, we investigated *VANGL2* variations in a particular birth cohort, the NFBCC1966, which has the advantage of being a very homogeneous population, studied in young adulthood (all subjects aged 31). This is a time when genetic influences tend to play a more important role compared with older ages, at which point such influences may be masked by the effects of cumulative exposure to lifestyle and environmental risk factors.

Cigarette smoke induces damage to lung epithelial cells along with redistribution of the actin cytoskeleton (Olivera et al., 2010) and reduced FVC is associated with decreased elastance, thus mirroring phenotypes found in *Vangl2^Lp/+^* mice. The interaction between SNPs and smoking that we observed results in a decrease of 5.1 ml per allele and per pack-year and, over time, this would considerably decrease total lung capacity. In line with our findings that *Vangl2* is required to maintain normal alveolar architecture, the observed *VANGL2*×smoking interactive effect on FVC could be explained by higher susceptibility of individuals with *VANGL2* variations to structural damage of the lung in response to noxious exposures such as smoking (Walter et al., 1979; Anderson et al., 1965). It is particularly notable that the effect of the *VANGL2* rs4656907 polymorphism on FVC was only observed through its interaction with smoking i.e. following exposure to a damaging insult, while neither the SNP nor smoking showed any effect on their own.

It is important to note that, unlike in mouse mutants, SNPs identified from human genome-wide data, such as those used here, are often only genetic markers, being typically correlated (in linkage disequilibrium) with the true causal variant (disease-causing polymorphism). Follow-up investigation, for example with full gene sequencing, is usually required to identify the true causal variant underlying the observed genetic association. It is interesting that the SNP we identify as modifying lung function in smokers is correlated with one in exon 8 – the same exon where the Looptail mutation lies in the mouse. It is possible that the impact of variations in a gene such as *VANGL2*, which is important in tissue repair, will change throughout life. It would therefore be interesting to carry out more extensive epidemiological studies in population cohorts of different ages.

### Vangl2 and alveologenesis

Embryonic *Vangl2^Lp/+^* mice show altered Vangl2 protein localization and extensive lung airspace enlargement, which becomes particularly apparent once the most active phase of alveologenesis is complete (P2 to P15 in mouse). These architectural defects are accompanied by elevated *Mmp12* gene expression and altered elastin deposition. Secretion of basement membrane components by fibroblasts and elastin deposition at the tips of migrating epithelial cells are both important for septation during the saccular and alveolar phases of development. Therefore, we propose that, during septation, deficits in both cell migration and elastin deposition contribute to the postnatal abnormalities identified in *Vangl2^Lp^* and these, combined with the earlier embryonic lung defects, lead to the severe airspace enlargement observed at 10 weeks. Elastin facilitates expansion and recoil of lung tissue during respiration and therefore the changes in elastance detected in *Vangl2^Lp/+^* mice are consistent with the structural defects observed. Elastin disruption is also associated with altered lung architecture and function in emphysema and this is known, in part, to result from increased MMP12 release from macrophages (Hautamaki et al., 1997), so it is particularly interesting that *Vangl2^Lp/+^* lungs show elevated *Mmp12* as well as differences in macrophage populations. Of note, Vangl2 has been shown to regulate Mmp2 and Mmp14, and remodelling of the ECM in other systems (Williams et al., 2012; Cantrell and Jessen, 2010). We did not detect a difference between the 45 kDa active Mmp12 protein levels in lung tissue; however, we were unable to reliably detect and quantify all forms of Mmp12 simultaneously using currently available anti-Mmp12 antibodies. In addition, since macrophages are the major cell type producing Mmp12, western blotting may not be sensitive enough to detect changes in whole lung lysate where macrophages comprise a relatively small proportion of the total cell population. Thus, we cannot completely rule out the possibility that there may be a difference in Mmp12 protein levels between WT and *Vangl2^Lp/+^* that we were unable to detect.

### Vangl2 and macrophage polarization

Tissue damage can affect both macrophage numbers and polarization, for example, emphysema patients show an increase in alternatively activated (M2) macrophages (Shaykhiev et al., 2009). Macrophages undergo specific differentiation depending on their local environment and these ‘activated’ macrophages have been categorized into two major subtypes, M1 and M2. In *Vangl2^Lp/+^* lungs, we found an increase in M2 macrophages, which are associated with immunosuppression and tissue repair (Mills, 2012), whereas genes associated with classically activated (M1) macrophages, linked to inflammation, are reduced. The increased number of M2 activated macrophages in *Vangl2^Lp/+^* mice may be due to an accumulation of tissue damage in the lungs that results from the developmental defects. In addition to their functions in inflammation and repair, macrophages are increasingly recognised to play an important role in tissue morphogenesis. For example, in the lungs, M2 type macrophages have recently been associated with tissue remodelling during alveolar formation (Jones et al., 2013). To our knowledge, this study is the first to show a link between the PCP pathway and macrophage biology in any non-malignant environment. Given the key relationship between macrophage activation and the balance between tissue damage and repair, it will be important to follow up these findings in future studies of the PCP pathway.

In summary, we propose that in the absence of an intact PCP pathway, alveolar formation is disrupted due to impaired cell migration, resulting in structural and functional defects and increased susceptibility to tissue damage/injury in the adult lung. Our data reveal that in addition to being a key pathway for embryonic development, the PCP pathway is also important for adult homeostasis and repair in the lungs. Importantly, manipulation of the PCP pathway may provide novel therapeutic approaches to enhance or promote tissue repair in degenerative lung diseases.

## MATERIALS AND METHODS

### Reagents

General laboratory reagents were purchased from Life Technologies (UK) unless otherwise stated.

### Mice

All animal maintenance and procedures were carried out according to the requirements of the Animal (Scientific Procedures) Act 1986. Surgery was performed under ketamine and sodium pentobarbital anaesthesia. Animal work was approved by the MRC Harwell AWERB committee. Lung function experiments were conducted at Imperial College and were approved by the South Kensington and St Mary's AWERB committee.

*Looptail* mice carry a point mutation at position 464 that causes a serine to asparagine amino acid change resulting in loss of function (Kibar et al., 2001; Murdoch et al., 2001). Mutant and WT mice were housed together in identical conditions. Mice were maintained on a C3H/HeH background (MRC Harwell, Oxford), housed in specific pathogen-free conditions and given food and water *ad libitum*; WT littermates were used as controls.

### Human material

Human embryonic and fetal material was provided by the Joint MRC/Wellcome Trust (099175/Z/12/Z) Human Developmental Biology Resource (www.hdbr.org). Normal adult lung tissue for immunostaining was obtained from the International Institute for the Advancement of Medicine (USA). Cohort 1 lung tissue was obtained from the Comprehensive Pneumology Center (CPC) Biobank (ethical approval by the University of Munich, 333-10) from COPD patients classified as Global Initiative for Chronic Obstructive Lung Disease (GOLD) IV undergoing lung transplant due to their underlying COPD and control subjects (transplant donors), *n*=6 per group. Cohort 2 lung tissue was obtained from the Biobank of the Respiratory Biomedical Research Unit (BRU), Royal Brompton and Harefield NHS Foundation Trust (ethics reference number 10/H0504/9). Control lung was obtained from patients undergoing surgical resection for suspected lung tumours and comprised histologically normal parenchymal tissue not directly involved in the excised lung cancer. COPD samples comprised emphysematous parenchymal lung tissue obtained from patients undergoing lung volume reduction surgery for severe radiological emphysema, *n*=8 per group.

### Antibodies

The following primary antibodies were used for immunohistochemistry (IHC), immunofluorescence (IF) and western blots (WB): rabbit anti-Vangl2 (Aviva Systems Biology, San Diego, CA, OAAB15535; 1:200, IHC), rabbit anti-Vangl2 (Ramsbottom et al., 2014) (21st Century Biochemicals, Marlborough, MA; 1:2000, WB), rabbit anti-Gapdh (Millpore, Temecula, CA, ABS16; 1:30,000, WB), rabbit anti-cofilin (Cell Signaling, Beverly, MA, 5175; 1:1000, WB), rabbit anti-p-cofilin (Ser3) (Cell Signaling, 3313; 1:1000, WB), rabbit anti-β-catenin (Cell Signaling, 9562; 1:1000, WB), mouse anti-β-actin (MP Biomedicals, 08691001; 1:10,000, WB), mouse anti-E-cadherin (BD Biosciences, 610181; 1:2500, WB), rabbit anti-GM130 (Sigma-Aldrich, G7295; 1:1500, IF), rabbit anti-elastin (Biorbyt, orb13391; 1:100, WB), rabbit anti-MMP-12 (Abcam, Ab52897; 1:1000, WB and 1:200, IHC), Armenian hamster anti-Pecam1 (Abcam, Ab119341; 1:500, IF; a gift from Leo Carlin), rabbit anti-phospho-histone H3 (Ser10) (PH3; Millipore, 06-570; 1:1000, IF), rabbit anti-cleaved caspase 3 (D175) (Cell Signaling, 9661; 1:1000, IHC), goat anti-CC10 (T-18) (Santa Cruz Biotechnology, Dallas, TX, sc-9772; 1:1000, IHC), rabbit anti-proSP-C (Millipore, AB3786; 1:1000, IHC), rabbit anti-non-phospho (active) β-catenin (Cell Signaling, 8814; 1:1000, WB).

### Histology

Lung lobes were inflated and fixed in 10% neutral buffered formalin (Sigma-Aldrich), embedded in paraffin and sectioned at 4 µm thickness. Sections were stained with Haematoxylin and Eosin, Miller's elastin stain and Mauritius Scarlet Blue (MSB) using standard histological methods.

### Immunostaining

Paraffin sections were de-waxed and incubated in primary antibody overnight at 4°C. Primary antibodies were detected using the Vectastain Elite ABC kit (Vector Labs, UK) and diaminobenzidine detection (BD Bioscience, UK) following the manufacturers' instructions. Antigen retrieval was performed for Vangl2, phospho-histone H3 and CC10 staining using citrate buffer (pH 6) incubation in a microwave for 3×3 min. Cryosections were incubated with Rhodamine-conjugated Phalloidin (Biotium, Hayward, CA, 00027; 1:200) following the manufacturer's instructions.

Lung slices were fixed in 4% paraformaldehyde (PFA) for 15 min, and permeabilized in 0.5% Triton X-100 for 30 min. Non-specific protein binding was blocked for 1 h at room temperature using 1% BSA and 0.2% Triton X-100 in PBS (PBS-BT). Slices were incubated with primary antibody overnight at 4°C, washed several times in PBS then incubated with donkey anti-rabbit-conjugated Alexa Fluor 594 (Life Technologies, A21207; 1:500) or goat anti-Armenian-Hamster-conjugated Cy3 (Jackson ImmunoResearch, West Grove, PA, 127-165-099; 1:500) secondary antibodies, and imaged using a Zeiss LSM-510 inverted confocal microscope. Controls, where the primary antibody was omitted, were included in every immunostaining experiment.

### DAB quantification

DAB intensity was quantified using the Fiji open-source programme (Schindelin et al., 2012). Images were captured in a tagged image file format (TIFF) and converted to an 8-bit image. Using a modification of Brey et al. (2003), image threshold was adjusted to highlight areas of DAB-positive staining with areas of high DAB intensity appearing first, and the same threshold was set for all images in the analysis (control MO versus VANGL2 MO). The percentage of the highlighted area above the set threshold was calculated using the built-in analysis tool. Quantification was performed from three independent experiments, each containing three technical replicates, from which two fields of view were assessed for control and VANGL2 knockdown.

### Protein extraction and western blot

Whole lungs from E14.5 mice or caudal lobes from 10-week-old mice were lysed in cell lysis buffer (Cell Signaling) supplemented with complete EDTA-free protease inhibitors (Roche), PhosSTOP phosphatase inhibitors (Roche), 1 mM PMSF (Sigma-Aldrich) and 50 mM NaF (Sigma-Aldrich). Subcellular fractionation to isolate the membrane and cytoplasmic fraction was performed using a Plasma membrane Protein Extraction Kit (Abcam) following the manufacturer's instructions. Protein concentration was assessed by Bradford assay (Sigma-Aldrich) following the manufacturer's instructions. 10 µg total protein was resolved using NuPAGE 4-12% BisTris gels, and transferred to nitrocellulose using an iBlot Gel Transfer Device (IB1001). After blocking with either 5% BSA (Sigma-Aldrich) or non-fat dried milk powder in 0.1% Tween-20 in TBS (pH 7.4; 20 mM Tris-HCl, 137 mM NaCl) for 1 h, membranes were incubated with primary antibody overnight 4°C, followed by either goat anti-rabbit IgG-HRP (Cell Signaling, 7074; 1:2000) or donkey anti-mouse IgG-HRP (R&D systems, HAF018; 1:3000) where appropriate. Detection was by ECL plus substrate (Thermo Fisher Scientific) following the manufacturer's instructions.

### Quantification of number of airways and airspaces

The number of airways and airspaces visible per field of view under a 20× magnification (0.19 mm^2^) was counted. The mean was calculated from counting the total numbers present in three fields of view per section at E18.5 and two fields of view per section at E14.5. Six separate sections were analysed per individual mouse.

### Mean linear intercept

Mean linear intercept (*L*_m_) of inflation-fixed left lung lobes was calculated following the method of Andersen et al. (2012). A grid of eight horizontal lines was superimposed on images of H&E stained sections, taken at 20× magnification, using Fiji and the Grid Overlay plug-in (written by Wayne Rasband). The number of times alveoli intercepted the line was counted and *L*_m_ was calculated using the following equation: *L*_m_=*NL/X*, where *N*=number of lines counted, *L*=length of line, and *X*=total number of intercepts counted. A minimum of three fields per lung section, and 12 sections per individual mouse were imaged. Fields of view containing blood vessels or airways were omitted from analysis.

### Quantification of elastin area in secondary crests

Left lung lobe sections from 10-week-old WT and *Vangl2^Lp/+^* mice were stained with Miller's elastin stain. The area of elastin deposition (dark blue stain) at the tips of secondary crests was quantified by drawing around the deposition area using the freehand tool in Fiji.

### Collagen Sircol assay

Protein was isolated as described above and total collagen levels were assessed by Sircol assay (Biocolor) following the manufacturer's instructions.

### RNA extraction and quantitative real-time PCR

Accessory lobes from 10-week-old adult mice were homogenised in Trizol and total RNA was extracted using an RNeasy mini kit (Qiagen, UK) following the manufacturer's instructions. 1 µg total RNA was converted to cDNA using a High-Capacity cDNA reverse transcription kit. Quantitative real-time PCR was carried out using TaqMan Fast Master Mix and TaqMan primers targeting *Col1a1* (Mm00801666_g1), *Col3a1* (Mm01254476_m1), *Eln* (Mm00514670_m1), *Fn1* (Mm01256744_m1), *Mmp2* (Mm00439498_m1), *Mmp9* (Mm00442991_m1), *Mmp12* (Mm_00500554_m1), *Nos2* (Mm00440502_m1), *Irf5* (Mm00496477_m1), *Fizz1* (Mm00445109_m1), *Mrc1* (Mm00485148_m1), *Pecam1* (Mm01242584_m1), *Tie2* (Mm00443243_m1), *Vegfr1* (Mm00438980_m1), *Pdgfra* (Mm00440701_m1), *VANGL1* (Hs01572998_m1), *VANGL2* (Hs00393412_m1), and *SCRIB* (Hs01034944_m1) from Life Technologies. Relative levels of gene expression were determined using Applied Biosystems Viia 7 Real-Time PCR System. Gene levels were normalized to the average of the housekeeping genes *B2m* (Mm00437762_m1) and *Hprt* (Mm01545399_m1) for murine studies. For human patient studies, genes were normalized to *B2M* (Hs99999907_m1). For A549 cells, gene expression was normalized to the average of the housekeeping genes *GUSB* (Hs00939627_m1) and *B2m*. Housekeeping genes were selected according to those that were most stable across experimental tissue samples following preliminary experiments. *Axin2* and *Nkd1* qRT-PCR analysis was performed using SYBR Green (Roche) and a LC480 Light Cycler (Roche) and normalized using *Hprt*. Forward and reverse primers used for qRT-PCR are listed in Table 2. Primers were used at a final concentration of 500 nM, with annealing temperatures between 56°C and 60°C.

**Table 2.**
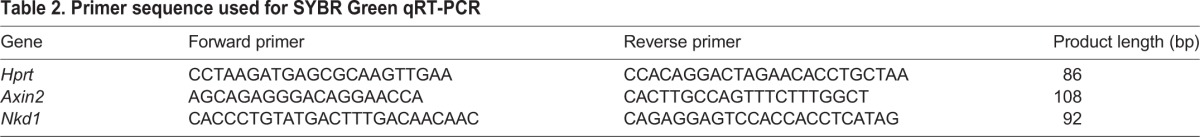
**Primer sequence used for SYBR Green qRT-PCR**

### Scratch assay

Human alveolar adenocarcinoma A594 cells (obtained from ATCC, 2015) were seeded at 50,000 cells/well in eight-well chamber slides (Thermo Scientific), and cultured for 72 h in Dulbecco's minimum essential medium (DMEM) supplemented with 10% foetal bovine serum (FBS) and penicillin and streptomycin (Life Technologies). For VANGL2-knockdown studies, cells were cultured with either VANGL2 (GTC CGA ACG CCG TGC CTT GTA GC), or control (CCT CTT ACC TCA GTT ACA ATT TAT A) antisense morpholino (MO) (GeneTools, Philomath, OR) at a final concentration of 10 µM. After 72 h of MO treatment, a single scratch was made through the centre of each well using a p1000 pipette tip. For VANGL2-knockdown studies A549s were then cultured for a further 24 h in culture medium supplemented with 10 µM of the appropriate MO. For WNT5A stimulation studies, A549 cells were cultured with DMEM supplemented with 0.5% FBS, penicillin and streptomycin, and 1 µg/ml recombinant WNT5A (R&D Systems) for a further 18 h. Wells were imaged using a Zeiss Axiovert 200 microscope at *t*=0 and *t*=24 h for VANGL2 knockdown, and at *t*=0 and *t*=18 h for WNT5A stimulation study. The area of the wound healed was calculated by drawing around the outline of the scratch using the freehand tool in Fiji and subtracting the area at *t*=24 or *t*=18 from the area at *t*=0 h.

For siRNA experiments, A549 cells were treated with 5 nM ON-TARGETplus SMARTpool VANGL2 siRNA (Dharmacon) or ON-TARGETplus Non-targeting Pool for 72 h for knockdown. Cells were then scratched using p200 tips and cultured for 18 h. Wells were imaged using JuLi Stage real-time cell recorder (NanoEnTek Inc.) at *t*=0 and *t*=18 h.

### Golgi orientation assay

Cell directionality during migration was quantified as previously described (Caddy et al., 2010). A cross with a 120° arc facing the direction of migration was placed on the nucleus of individual cells at the leading edge. Golgi were considered polarised if they were present within the 120° arc facing the direction of migration. Golgi orientation was quantified from three individual experiments, with each individual experiment containing three technical replicates, and from each technical replicate at least five fields of view along the leading edge was quantified for control and VANGL2 knockdown.

### *Ex vivo* lung slice culture

Lung slices were obtained using a modification of the method described (Henjakovic et al., 2008). Briefly, lungs from 11- to 13-week-old mice were inflated by intratracheal administration of 1.2 ml 2% low melting point agarose (Sigma-Aldrich) and allowed to cool. The left lung lobe was isolated and embedded in 2 ml 2% agarose and sliced at a thickness of 300 µm using a tissue slicer (Precisionary Instruments, San Jose, CA). Slices were incubated in DMEM containing penicillin and streptomycin for 1 h at 37°C, 5% CO_2_ and then replaced several times with fresh medium prior to overnight incubation to remove excess agarose prior to fixation in 4% PFA.

### Respiratory mechanics measurements

Respiratory mechanics were measured in 10-week-old male WT and *Looptail* (*Vangl2^Lp/+^*) mice using the forced oscillation technique (Flexivent system, SCIREQ, Canada) in anaesthetized, ventilated mice. Following lung function measurements, BALF and lung tissue samples were collected and processed based on the method by McMillan et al. (2005). Left lung lobes were inflated and fixed for histology as detailed above. The remaining lobes were snap frozen for further analysis.

BALF was centrifuged for 30 s to pellet cells, and the supernatant stored for further analysis. The cell pellet was resuspended in 0.5 ml RMPI supplemented with 10% FCS and penicillin and streptomycin. 50,000 cells were then loaded onto a cytospin and spun at 400 rpm for 4 min using a Shandon Cytospin3 (GMI Inc., Ramsey, MN, USA), and fixed in methanol for 5 min. Cells were then stained using Wright-Giemsa stain (Sigma-Aldrich) following the manufacturer's instructions. Immune cell populations were assessed by morphology by counting 300 cells per sample. The enlarged vacuolar macrophages were characterised by morphology, normal macrophages were defined as small rounded cells, whereas enlarged macrophages were two or three times larger in size and more granular.

### Study on the Northern Finland Birth Cohort 1966 (NFBC1966)

We investigated whether variants in the *VANGL2* gene can affect lung function and whether they can modify susceptibility to the effects of smoking, a common cause of lung tissue damage, by testing the effect of smoking×*VANGL2* interactions on FVC and on FEV_1_/FVC in the NFBC1966. This is a population-based birth-cohort study that recruited pregnant women with an expected date of birth in 1966 in the provinces of Oulu and Lapland, Finland (Rantakallio, 1988). The analyses were based on 5139 subjects for whom genetic and spirometry data were available.

On all subjects, spirometry was performed three times using a Vitalograph P-model spirometer (Vitalograph), and was repeated if the coefficient of variation between the two maximal readings was >4%. Pack-years of smoking were calculated by multiplying the number of packs smoked per day by the number of years the subject had smoked. A total ‘smoke pack’ per day was calculated assuming that one smoke pack was equivalent to 20 filter cigarettes or 10 other cigarettes or 8 pipefuls or 5 cigars.

For *VANGL2*, 14 SNPs were identified for which good-quality imputed data (HapMap 22 release) were available in NFBC1966, after excluding SNPs with MAF<0.05 or in complete linkage disequilibrium with others in our population (*r*^2^>0.99). The 14 SNPs were: rs11265388, rs7513382, rs12083518, rs12564849, rs6427518, rs11586476, rs11265386, rs4656906, rs12088083, rs4656907, rs16831953, rs12090877, rs11265393, rs6663715.

### Statistical methods

#### Analysis of experimental data

Data were analysed using Prism 5 (GraphPad). Unless otherwise stated, graphs show the mean values for each experimental group with variation represented as s.e.m. Differences between groups were tested using a two-tailed Student's *t*-test, with a threshold for statistical significance of 0.05.

#### Analysis of human population-based data

Owing to the problem of multicollinearity when analysing the 14 SNPs together in a classical regression model, we used elastic-net regularization. This is a variable selection method to identify variables in the model (e.g. SNPs and SNP×smoking interaction terms) with statistical support from the data, which works well when the model contains highly correlated variables as in our case (Zou and Hastie, 2005). For both FVC and FEV_1_/FVC ratio, modification of the effect of smoking by *VANGL2* polymorphisms was assessed by including in the linear model the interaction terms between each SNP and pack-years, along with the SNPs and pack-years (main effects) and adjusting for sex, height and the first two ancestry principal components to control for population stratification. Age was not included in the model since the study is a birth cohort with all subjects aged 31 years. We implemented the model with a regularization parameter (λ) based on cross-validation and an elastic-net penalty (α) of 0.5, using the *glmnet* package in R (Friedman et al., 2010). Estimates of main and interaction effects for those SNPs showing interaction in the elastic-net model were obtained from a linear regression model including the selected SNP, smoking, SNP×smoking, sex, height and two principal components. These effect estimates from the classical regression model and their 95% confidence intervals should be interpreted as indicative, given that they do not account for the variable selection process performed in the elastic-net analysis and may be slightly overoptimistic.
